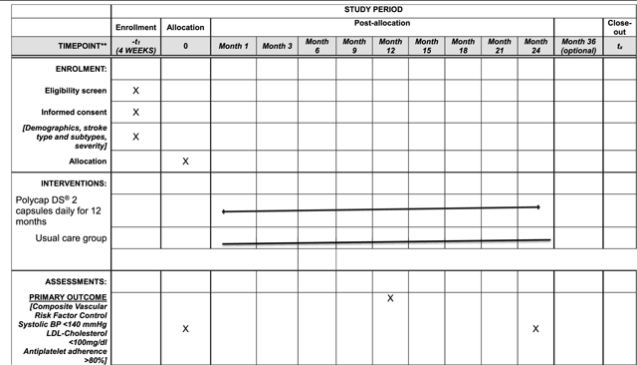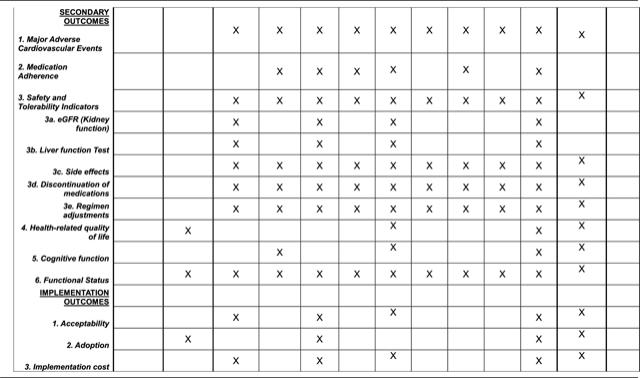# Stroke minimization through additive anti-atherosclerotic agents in routine treatment (SMAART) II: Rationale for a multi-country polypill phase 3 trial in sub-Saharan Africa

**DOI:** 10.1016/j.neuros.2026.100020

**Published:** 2026-01-22

**Authors:** Fred Stephen Sarfo, Kolawole Wahab, Sarah Shali Matuja, Philip Babatunde Adebayo, Thierry Adoukonou, Raelle Tagge, Bruce Ovbiagele

**Affiliations:** a Kwame Nkrumah University of Science and Technology, Kumasi, Ghana; b Komfo Anokye Teaching Hospital, Kumasi, Ghana; c University of Ilorin Teaching Hospital, Ilorin, Nigeria; d Catholic University of Health and Allied Sciences, Weill Bugando, Tanzania; e Aga Khan University, Dar es Salaam, Tanzania; f University Teaching Hospital of Parakou, Parakou, Benin; g North Californian Institute of Research & Education, USA; h University of California San Francisco, USA

**Keywords:** Africa, Atherosclerosis, Polypill, Secondary prevention, Stroke

## Abstract

**Rationale:**

Global estimates suggest that sub-Saharan Africa (SSA) now has the highest incidence, prevalence, and worst survival outcomes of stroke. Fixed-dose combination pills, also known as “polypills”, containing generic drugs: Aspirin, a statin, and blood pressure (BP) lowering medications may be a viable low-cost avenue to broadly improve medication adherence and consequently reduce further disability or death on a large scale among stroke survivors in SSA.

**Objectives:**

The Stroke Minimization through Additive Anti-atherosclerotic agents in Routine Treatment II *(*SMAART-II*)* study seeks to deploy a hybrid study design to *1)* demonstrate the efficacy of a polypill (Polycap ^®^) containing fixed doses of antihypertensives, a statin, and antiplatelet therapy taken as *two capsules*, once daily orally in reducing composite vascular risk over 24 months vs. usual care among 1000 recent ischemic stroke patients encountered at primary and tertiary hospitals in Benin, Nigeria and Tanzania; *2)* develop an implementation strategy for routine integration and policy adoption of polypill for post-stroke cardiovascular risk reduction in an under-resourced system burdened by suboptimal care and outcomes.

**Expected impact:**

SMAART II will establish the definitive efficacy and safety of the polypill to improve meaningful post-stroke global risk factor control across several sites, across diverse healthcare settings, beyond tertiary level care, and over a longer period. In addition to assessing clinical outcomes, SMAART II will assess implementation outcomes such as adoption, acceptability, cost, pertinent to uptake of the polypill strategy in the three proposed African countries to inform policy.

**Trial registration:**

NCT05963568

## Introduction

Sub-Saharan Africa (SSA) now bears the heaviest burden of stroke worldwide, with an age-standardized stroke incidence of 316 per 100,000, and prevalence rate of 1.4 per 1000 population. Outcomes of stroke in SSA are abysmal with 1-month case fatality at 30 % and 3-year mortality rate of 84 % [1–8]. Stroke survivors in SSA are at an inordinately high (and worsening) risk of adverse outcomes over the medium-to long-term [6,8,9]. The most recent iteration of the Global Burden of Disease study reported stroke prevalence of 2 per 1000 in Ghana, the highest reported prevalence on the globe [10].

Stroke survivors in SSA are especially at high risk for recurrent vascular events or death due to several factors, including uncoordinated health systems, under-controlled vascular risk factors [11], and lack of care affordability. Secondary prevention guidelines recommend that antihypertensive, statin and antiplatelet therapy should be initiated promptly after ischemic stroke and adhered to in a persistent fashion to achieve optimal vascular risk reduction [12–16]. However, these goals are seldom realized in routine clinical care settings, especially in SSA [17–26]. Of note, the Neurologist-population ratio in SSA ranges from 1 per 162,885 persons to none in 11 countries (vs. 1 per 29,200 persons in the US) [27], underscoring the need to identify relatively simple strategies that can be applied broadly for stroke survivors in resource-constrained settings. Fixed-dose combination pills, also known as “polypills”, containing generic drugs: aspirin, a statin, and blood pressure (BP) lowering medication(s) may be a viable low-cost avenue to broadly improve medication adherence and consequently reduce further disability or death on a large scale among stroke survivors in SSA [28–42]. Importantly, a significant majority of stroke survivors in Africa receive healthcare from primary care settings with limited to no access to specialist physicians [43]. Therefore simplified, cost-effective, secondary prevention strategies for post-stroke care that could be safely implemented by non-specialist physicians to the millions of stroke patients across Africa and indeed resource-constrained low-income countries is an urgent priority.

Five major international randomized controlled trials (RCTs) on polypills powered to assess major adverse cardiovascular events have been completed or are currently on-going. Two of these-The International Polycap Study 3 (TIPS-3) [44], and HOPE-3 [45], were primary prevention studies, two others- the Preventative Role of a fixed dose combination Pill in Stroke (PROPS) [46] and the Secondary Prevention of Cardiovascular Disease in the Elderly Trial (SECURE) [47] are secondary prevention trials and PolyIran study [48] aimed at both primary and secondary CVD N prevention. The key outcomes of these studies are summarized in Supplementary Table 1 which demonstrates the efficacy of the polypill for both primary and secondary prevention of Atherosclerotic Cardiovascular diseases (ASCVDs). We conducted a phase 2 surrogate biomarker outcome and safety clinical trial (SMAART-1) comparing effects of a polypill containing fixed doses of 3 antihypertensive agents, a statin and antiplatelet taken daily for 12 months vs. usual care at the Komfo Anokye Teaching Hospital, a single tertiary medical center in Ghana [49]. The mean change in Carotid Intimal Media Thickness at month 12 from baseline was − 0.017 ± 0.26 mm in the polypill arm vs − 0.092 ± 0.18 mm in the usual care arm. The mean difference between the 2 arms of 0.049 mm (95 % CI: −0.008 – 0.109), p-value of 0.105 using analysis of covariance accounting for baseline differences between the two arms. Cost effectiveness analysis show that the polypill is a promising and potentially cost-effective intervention which should be incorporated into a multi-pronged and holistic strategy for prevention of atherosclerotic cardiovascular diseases (ASCVD) in LMICs [50,51]. To date, there are no phase 3 secondary prevention trials for stroke survivors using the cardiovascular polypill as a strategy for reducing global cardiovascular risk and clinically significant events such as recurrent strokes or other ASCVDs as a secondary prevention strategy.

## Potential study objectives and guiding frameworks

An overarching goal would be to establish the efficacy of a polypill (Polycap ^®^) in reducing composite vascular risk over 24 months vs. usual care among 1000 recent ischemic stroke patients encountered at hospitals in Benin, Nigeria, and Tanzania. The following would be the specific aims:

### Specific Aim #1:

To conduct a parallel group, non-inferiority, randomized clinical trial to assess the efficacy of the polypill vs. usual care in achieving blindly adjudicated composite vascular risk factor control (systolic BP <140 mm Hg, low density lipoprotein (LDL)-cholesterol < 100mg/dL, 80 % adherence to anti-platelet therapy) at month 12 and 24 as primary outcome measured among 1000 recent stroke survivors receiving care at 12 Beninese, Nigerian, and Tanzanian medical centers (primary, secondary, and tertiary-levels care).

**Hypothesis:** A polypill formulation comprising cheap generic drugs is not inferior to use of multiple single formulations for ischemic stroke survivors but is cheaper and safer with implications for the patient and health system in SSA.

### Specific Aim #2:

To assess the effectiveness of the polypill at improving incident major adverse cardiovascular events (fatal and non-fatal recurrent strokes, acute ST elevation myocardial infarction or nSTEMI, or cardiac deaths), all-cause mortality, medical regimen adherence, quality of life, and safety measures, including types and severity of adverse events over 24 months.

### Specific Aim #3:

To assess adoption, acceptability, and cost of the polypill strategy as implementation outcomes. We will identify context-specific implementation facilitators and barriers and devise a theoretically guided and evidence-based implementation strategy for routine use and policy adoption of the polypill in Benin, Nigeria, Tanzania, and Ghana through multiple stakeholder engagements.

### Guiding frameworks:

Lack of theoretical development has been proposed as a major contributor to failure to demonstrate efficacy of complex interventions in preventive care after stroke. As such, we anticipate that an effective intervention to improve outcomes among stroke patients in LMICs must be based in solid theoretical constructs tailored and relevant to the unique health care situation. The SMAART II conceptual model integrates key theoretical constructs and is the framework for organizing the intervention components. (Table 1)

## Potential study design, justification, and study sites

### Potential Study Design:

SMAART II would be phase III non-inferiority, randomized, open label, blinded endpoint clinical trial to evaluate the efficacy and implementation of a cardiovascular polypill (composed of fixed doses of 3 antihypertensives, a statin and anti-platelet) vs usual care (separate individual secondary preventive medications) for 24 months in achieving global CV risk reduction as primary outcome. This will be a 2-arm RCT involving 1000 stroke survivors with individual participant randomization.

### Justification for a non-inferiority randomized clinical trial design:

A polypill containing an antiplatelet, a statin, and antihypertensives has the potential advantage of improving medication adherence by stroke survivors via administration of fewer daily pills and improving compliance by clinicians to recommended guidelines at a relatively cheaper cost. However, because polypills are fixed dose combination pills, there is limited flexibility for dose adjustments and treatment-limiting side effects to a single component e.g. cough from ramipril, will lead to it withdrawal from further use. On the other hand, the standard of care involving individual, separate antihypertensive, statin and anti-thrombotic medications offers greater flexibility in dose adjustments and replacement of agents associated with side effects but comes at a higher pill burden with attendant loss of adherence and potentially higher costs.

There is therefore equipoise in assessing the clinical efficacy and safety of either approach for vascular risk factor control for secondary prevention after stroke. We have powered our study as a non-inferiority RCT to assess whether the polypill as an intervention is not worse than the standard of care at a non-inferiority margin of 7 % due to the reasons presented in the justification. This trial seeks to assess the polypill as viable alternative treatment approach for stroke survivors among with medication non-adherence issues.

#### Superiority end-point evaluation co-design:

We have also calculated a sample size required to achieve a superiority margin of 12 % in favor of the intervention i.e. the polypill over the standard of care over 24 months in global CV risk reduction. *The advantage of this approach would be able to answer the question of the polypills non-inferiority or superiority in a single trial, in resource-limited settings where funding for such a major undertaking is usually limited.* We will assess major clinical events such as recurrent strokes, CV deaths and safety measures as secondary outcome measures over 24 months with an optional follow-up at 36 months post-enrollment to assess additional longer-term vascular and cognitive outcome measures.

### Potential study Centres:

SMAART II will potentially recruit stroke cases from within a network of about 12 Beninois, Nigerian, and Tanzanian hospitals including primary and tertiary centers. Including stroke survivors from lower cadres of healthcare delivery in three African countries (West and East of Africa) will enhance the generalizability of our findings without comprising quality of clinical trial data. *The inclusion of lower cadres of healthcare in Benin, Nigeria, and Tanzania is to allow us to test the intervention among socio-economically deprived stroke patients*

## Study participants: inclusion/exclusion criteria

### Potential Participants:

1000 Beninese, Nigerian, or Tanzanian, adults’ patients with recent (within three to six months of symptom onset) stroke with uncontrolled hypertension meeting inclusion/exclusion criteria.

#### Inclusion Criteria:

1. Above the age of 18 years; male or female
2. Ischemic stroke diagnosis no greater than two months before enrollment. Ischemic strokes including lacunar, large-vessel atherosclerotic, embolic stroke of undetermined source subtypes are eligible
3. Participants with stroke may present with at least one of the following additional conditions: Documented diabetes mellitus or previous treatment with oral hypoglycemic or insulin; documented hypertension >140/90 mmHg or previous treatment with antihypertensive medications; Mild to moderate renal dysfunction (eGFR 60–30ml/min/1.73m^2^); Prior myocardial infarction
4. Legally competent to sign informed consent.

#### Exclusion Criteria:

1. Unable to sign informed consent;
2. Contraindications to any of the components of the polypill;
3. Hemorrhagic stroke;
4. Cardio-embolic stroke based on clinical evidence or EKG evidence of atrial fibrillation.
5. Severe congestive cardiac failure (NYHA III-IV);
6. Severe renal disease, eGFR <30ml/min/1.73m^2^), renal dialysis; awaiting renal transplant or transplant recipient;
7. Cancer diagnosis or treatment in past 2 years;
8. Need for oral anticoagulation at the time of randomization or planned in the future months;
9. Significant arrhythmias (including unresolved ventricular arrhythmias or atrial fibrillation);
10. Nursing/pregnant mothers;
11. Do not agree to the filing, forwarding and use of his/her pseudonymized data.

#### Potential Study protocol:

**Fig. 1.**
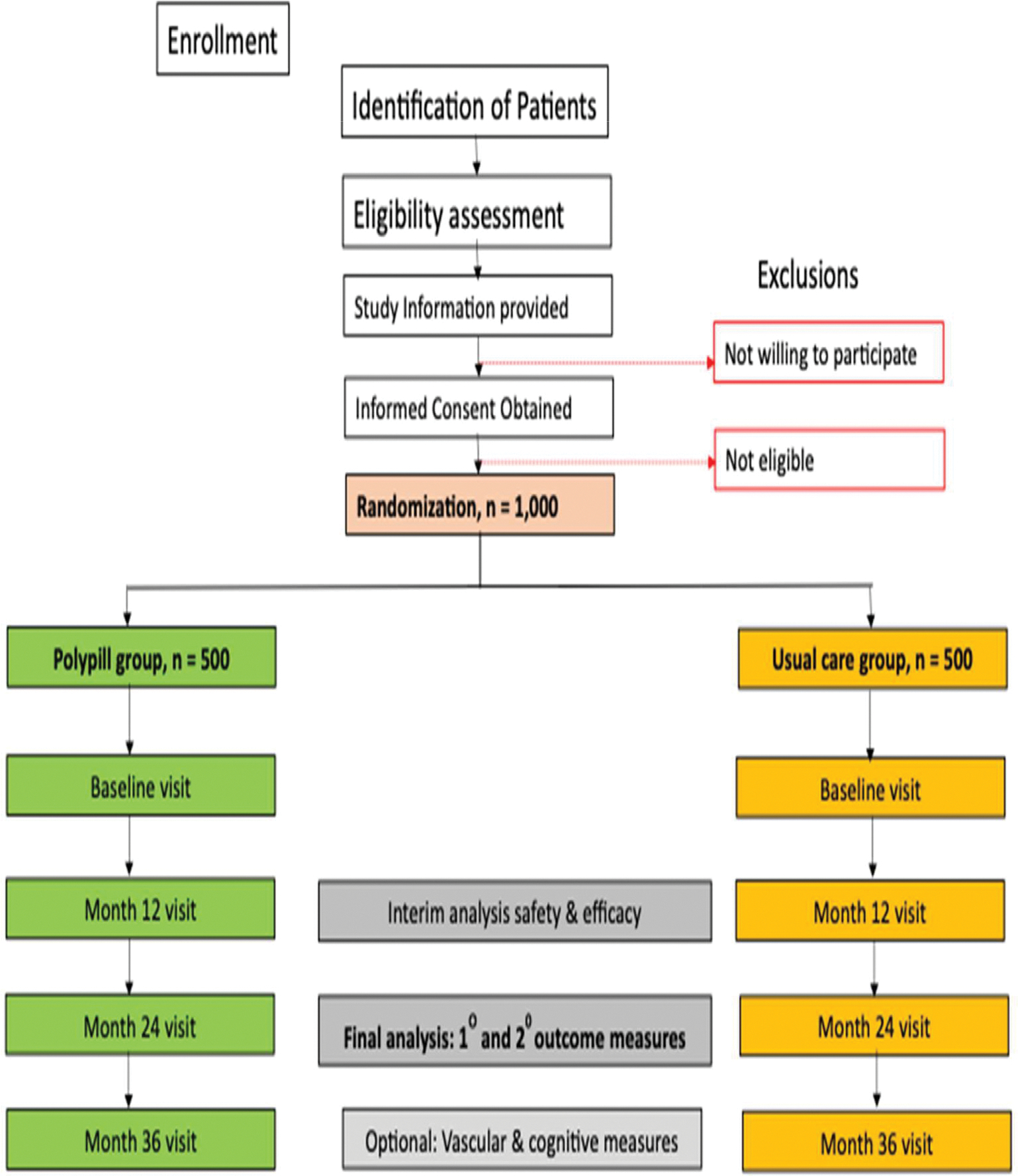
SMAART II Trial Flow Chart.

The diagnosis of stroke will be defined as an acute episode of focal cerebral dysfunction caused by infarction of central nervous system tissue, not resulting in death. Patients meeting eligibility criteria will be allocated to the experimental or active comparator arm. See Fig. 1 for SMAART II study design flow chart and Study Chart below (Table 2).

#### Randomization:

Once eligibility, consent and baseline data are confirmed, participants will be randomly assigned to polypill vs. usual care in a 1:1 allocation ratio, stratified by study site. An adaptive block randomization scheme will be developed and maintained by the lead study biostatistician. Once a patient is randomized he or she will be entered into the study and included in intent-to-treat analysis.

#### Masking and Blinding:

Due to the nature of the intervention, it is impossible to blind the patients, clinicians (nurses and doctors), and study coordinators to the group assignment of each intervention. Clinical outcome measures such as blood pressure will measured using an automated BP device which prevents study coordinator from influencing in any manner the BP readings; a centralized laboratory will measure lipid panels; and an adjudication panel will certify all major adverse cardiovascular events (MACE).

#### Intervention arm:

Patients allocated to the experimental arm will receive Two (2) (Polycap ^®^) taken orally once a day. A capsule of Polycap ^®^ contains 100 mg of Aspirin, 20 mg of simvastatin, 12.5 mg hydrochlorothiazide, 5 mg of ramipril and 50 mg of atenolol. Patients assigned to Polypill will have their antihypertensive agents, lipid modifiers and anti-thrombotic agents withdrawn and replaced with the polypill if they are already receiving such treatments before enrollment.

#### Control arm:

Patients allocated to the usual care arm will receive standard of care therapies for secondary prevention with drugs and doses left at the discretion of the treating physicians. Since our focus is to isolate the effect of the polypill strategy itself and create equipoise, at study inception providers for patients in both study arms will receive a brief one-time (Skype-based) training and a one-time email synopsis on guideline recommended biomarker targets after stroke.

#### Screening/run-in period-4 weeks:

All potentially eligible participants referred to or who contact the study team directly will undergo screening to determine eligibility using the pre-specified criteria.

#### Enrollment evaluation:

Information on stroke type from a cranial CT scan performed within 10 days of stroke symptom onset will be reviewed by Study Physicians, stroke subtype information where available will be sought to classify ischemic stroke using the TOAST classification [61] into cardio-embolic, large-artery and lacunar ischemic stroke. Stroke severity and functional status at enrollment will be assessed using the modified national institutes of health stroke scale (NIHSS) [62] and Modified Rankin Score [63], followed by a detailed assessment of vascular risk factors namely hypertension, diabetes mellitus, dyslipidemia, cigarette smoking, from history and physical examination. Blood samples for baseline assessments of renal, liver function tests, lipid profile and HBA1C will be collected and contraindications for study medications assessed. All concomitant medications will be recorded in the case report form.

#### Follow-up and management of possible treatment related side effects:

Participants will be followed for 24 months with scheduled visits at months 1, 3, 6, 9, 12, 18 and 24 for clinical assessments. Participants who experience side-effects will be reviewed by their physicians to assess severity and appropriate measures instituted. Co-morbidities such as diabetes will be managed following the Ghana Standard Treatment Guidelines.

### Potential Outcome and Mediator measures:

**Primary outcome** is proportion of participants in each arm achieving blindly adjudicated composite vascular risk factor control (systolic BP <140 mm Hg, LDL-cholesterol <100mg/dl, antiplatelet adherence by pill count >90 %) at month 12 and month 24 (sustainment of effect). This primary outcome measure was chosen because (i) they are physiological indicators of stroke risk that are the targets of secondary stroke reduction efforts, and intermediate steps to vascular event rate reduction, and (ii) they can be measured reliably in resource limited settings where this trial will be conducted.

### Possible Secondary outcomes:

#### Major Adverse Cardiovascular events:

include recurrent stroke which could be fatal/severely disabling stroke or non-fatal stroke; coronary artery disease: acute STEMI/NSTEMI, sudden cardiac deaths. MACE will be confirmed by a blinded adjudication committee by reviewing available clinical notes supported by investigations for example CT scans, EKGs, troponin tests, death certificates or verbal autopsy if death occurs outside hospital.

#### Change in adherence to therapy:

This will be measured at baseline, months 3,6,9,12,18 and 24 clinic visits using the self-reported Morisky-Green questionnaire (MAQ) [64] and pill count. Patients have to meet both criteria for adherence at the in-person visits to be considered adherent.

#### Safety and tolerability indicators:

- Renal function: Serum creatinine measurements to calculate eGFR using the CKD-EPI [65] formula at baseline and months 1, 6, 12 and 24.
- Liver function: Elevations in liver enzymes will be assessed at months 1, 6, 12 and 24. AST or ALT rises >5x will be considered significant elevations.
- Side effects profile: Adverse events will be closely monitored and side effects will be documented according to the NIH/NCI Common Toxicity Criteria [66]

#### Health related Quality of life:

The EQ-5D questionnaire [67] and the NINDS Neuro-QoL^™^ questionnaires will be used to assess state of health of study subjects at baseline, Month 12 and 24.

#### Power and sample size justification:

Our sample size calculation and power analyses are based on the proportion with composite outcome of systolic BP<140 mmHg, LDL-cholesterol <100mg/dl and antiplatelet therapy adherence >80 % at 12 (interim) and 24 months (final), as guideline recommended targets for vascular risk reduction targets after stroke. In the SMAART I pilot study by this group in Ghana, where this proposed study will take place, 148 subjects (74 per group) were randomized to polypill and usual care arms. The proportion with systolic BP<140 mmHg, LDL-cholesterol <100 mg and antiplatelet therapy adherence of >90 % at month 12 was *61.7 % in the polypill arm* vs *57.1 % in the usual care arm**, p* > 0.05.

For this phase 3 trial, we will enroll 680 participants meeting eligibility criteria to provide sufficient power to address our non-inferiority and superiority measures of intervention effect for the primary composite outcome. Power calculations for non-inferiority and superiority effect measures are shown below:

##### Power calculation 1:

A **non-inferiority margin** of difference between the group proportions set at 7 % (60 % in the polypill arm vs 53 % in the usual care arm) using a type I error of 0.05, a power of 90 %, and a one-sided significance level of 0.025 will require a minimum sample size of 526 subjects. Assuming a 20 % loss to follow up due to withdrawal or attrition and 20 % loss due to deaths over 24 months, the conservative target for enrollment is 880 to assess our primary outcome measure.

##### Power calculation 2:

A **superiority margin** of difference between the group proportions set at 12 % for the primary composite outcome measure at 24 months (60 % in the polypill arm vs 48 % in the usual care arm) using a type I error of 0.05, a power of 80 %, and a two-sided significance level of 0.05 will require a minimum sample size of 540 subjects. Assuming a 20 % loss to follow up due to withdrawal or attrition and 20 % loss due to deaths over 24 months; the conservative target for enrollment is 1000 to assess our primary and secondary outcome measures.

#### Potential Analytical Plan:

Descriptive statistics will be computed for all study variables, including average follow-up time, retention rate, protocol deviations and violations, and will be compared by treatment group. We will use *t*-test (Wilcoxon rank sum test) for continuous variables and chi-square test for categorical variables to make comparisons between two groups at baseline.

- **Primary analysis outcome:** The proportion in each arm achieving the composite global CVD outcome measure of systolic BP<140 mmHg, LDL-cholesterol <100mg/dl and antiplatelet therapy adherence >90 % at months 12 and 24 will be compared using chi-squared test. Non-inferiority of the polypill over usual care will be accepted if the lower bound of the 95 % CI around the estimated difference in the rate of global CV risk control rate lies above 8 %. The primary analyses will be performed according to the principle of intention to treat. Those who die, who is lost to follow-up or withdraws are assumed to have failed to achieve primary outcome measure. It is increasingly recognized that non-inferiority studies should be evaluated against a per protocol population defined on the basis of compliance, protocol violations, and missing data. Both sets of results are important and will be considered when assessing the primary outcome. In the case that non-inferiority is evident, assessment as to whether polypill is superior to usual care will be carried out using the same approach but comparing to a zero difference and applied to the intention to treat population.
- **Secondary outcomes**: Differences in time to events (for example MACE) between the two groups will be compared using a log-rank test and a Cox proportional hazards (pH) model to adjust for observed confounders and for clustering via frailty models. We will employ longitudinal data methods (generalized linear mixed models [GLMM]),^164^ which account for correlation of outcomes due to repeated measurements (for example BP, Cholesterol levels, medication adherence, quality of life) and missing at random (MAR) data to study the change in outcomes over time between the two groups. We will model binary (logit link & binomial distribution) and count outcomes (log link and poisson or negative binomial distribution) by properly selecting the link and distribution option of the GLMM.
- **Sensitivity analyses:** Additional analyses will assess patients’ adherence to secondary prevention therapies and biological variables, in particular, age of participants, sex (**a biologic variable of interest**), socio-economic factors, healthcare factors such as level of health facility on study outcomes.

## Assessment of implementation outcomes

To better appreciate the health system contexts within which the polypill would be used to manage stroke patients in Africa, it would be crucial to assess adoption, acceptability, and cost of the polypill strategy as implementation outcomes. Necessary to this goal would be to identify context-specific implementation facilitators and barriers and devise a theoretically guided and evidence-based implementation strategy for routine use and policy adoption of the Polypill in Benin, Nigeria, Tanzania, and Ghana through multiple stakeholder engagements.

This endeavor will be guided by the Normalization Process Theory (NPT), Community-based participatory research (CBPR) and NIH best practices for mixed methods research, to conduct a mixed methods study to identify implementation barriers and facilitators, understand the implementation context and propose theory- or framework-guided implementation strategies for the SMAART polypill intervention.

### Theoretical underpinnings for evaluating implementation outcomes:

A greater use of explicit theory in order to understand barriers and facilitators, select implementation strategies and design interventions, has been advocated to advance implementation research [68,69]. The Normalization Process Theory (NPT) is a widely used [58–60] theory of implementation that explains the process by which an intervention becomes, or indeed fails to become, normalized into routine practice [70,71]. NPT is represented by four constructs namely *coherence* (the work that people do to understand and make sense of a practice)*, cognitive participation (*the work that people do to engage and support a new practice)*, collective action (*the work that people do to enact a new practice, and make it workable and integrate it in its context)*, and reflexive monitoring (*the work that people do to reflect on and evaluate enacting a new practice in context). We will also be draw on principles derived from the work by Grol and Wensing [72] to provide a systematic approach to implementation planning for the SMAART polypill intervention.

**Fig. 2.**
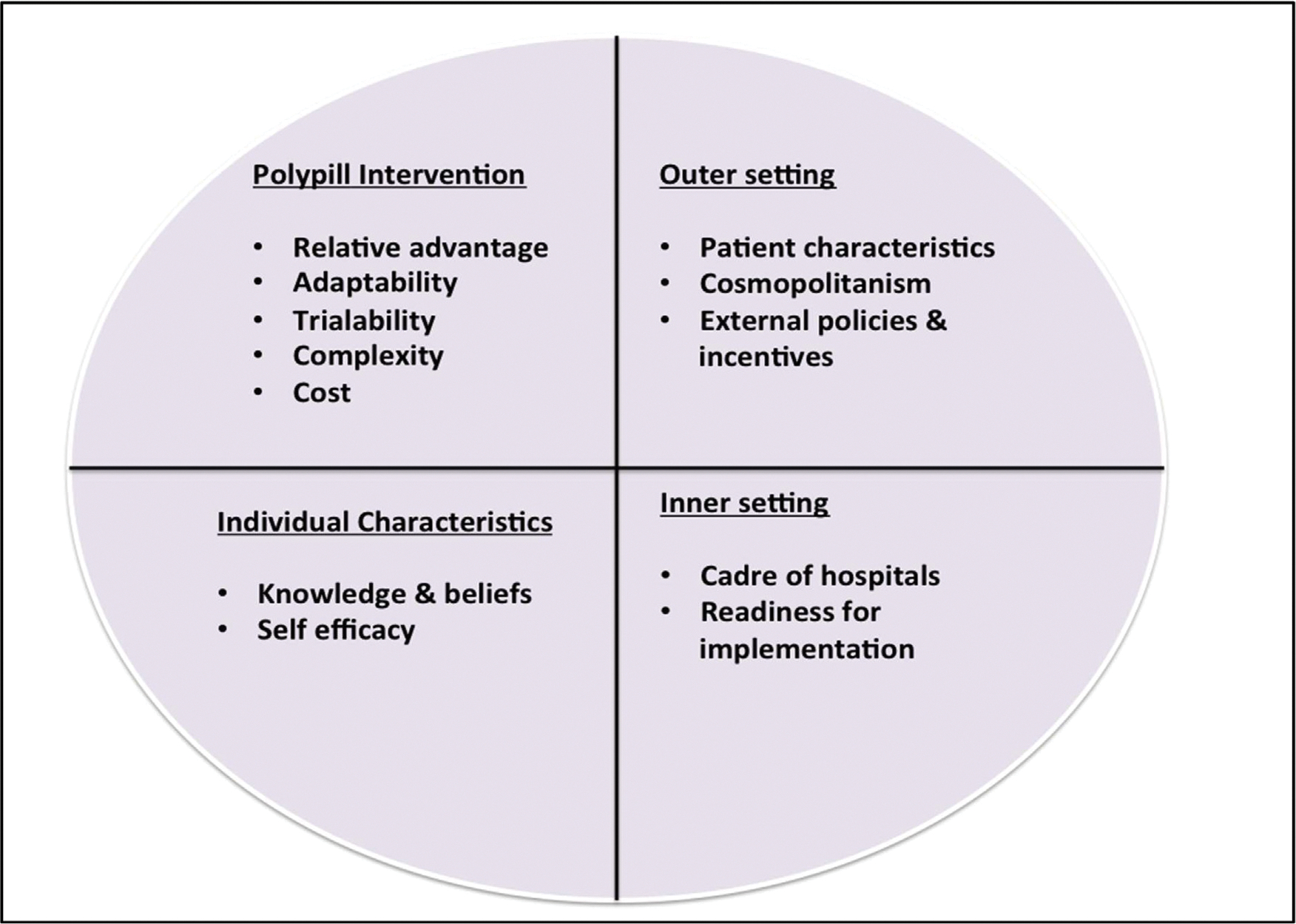
CFIR domains for Polypill Implementation.

The Research team will undertake a systematic literature review on factors that influence the implementation of evidence-based interventions for secondary prevention of CVDs after stroke in LMIC settings. Data from the review will be extracted and synthesized according to the Consolidated Framework for Implementation Research (CFIR) which categorizes implementation factors as relating to the intervention; the inner and outer contexts; the individual; or the process of implementation. (Fig. 2)

To understand the context for implementation of polypill into routine care in Benin, Nigeria, and Tanzania, we will engage key stakeholders including policy makers, implementers, Health providers at various cadres of service delivery and stroke survivors and family caregivers as end-users of the SMAART Polypill intervention. Question leads for stakeholder engagements will be guided by the CFIR assessing key domains of the framework namely intervention (polypill) characteristics, outer setting, inner settings and individual characteristics as shown in Fig. 2. We will employ implementation methods such as concept mapping and group model building which are inherently participatory approaches with the potential of galvanizing stakeholders around common goals and creating consensus on implementation barriers.

For each barrier identified at the patient-level (e.g. fear of side effects, cost of polypill), provider-level (for example, misconceptions such as inability to titrate polypill dosages or add-on other therapies), or at health-system and policy-level (for example engagement with pharmaceutical companies for high volume, low margin sales, inclusion of polypills into the national essential medicines list for insurance coverage), we will propose implementation strategies following guidelines proposed by Proctor et al. [73].

### Potential procedures:

This use of focus groups (FGs) of 8–12 participants per study sites followed by key interviews could be deployed. The FGs will last 60–90 min and the KIIs will last approximately 45–60 min and will occur at a location convenient to participants. A trained moderator will conduct the sessions with another member of the investigative team taking field notes during the session. Written consent will be obtained from participants prior to the start of any session. The purposive sample (based on demographics of the stroke survivors assigned to the Polypill intervention) for the FGs and KIIs (based on the clinical care providers (MDs, nurses, pharmacists, hospital administrators, policy makers) will be recruited. **Survey data** could also be collected through self-filled questionnaires to be completed by stroke survivors on polypill intervention (*n* = 250) and healthcare workers involved in post-stroke care (*n* = 50 from primary, secondary and tertiary medical facilities), health administrators and policy makers (*n* = 10) after obtaining informed consent.

### Implementation Outcomes:

Among the 500 subjects assigned to the SMAART intervention, the use surveys, qualitative or semi-structured interviews, checklists could be used to assess **three (3)** implementation outcomes proposed by Proctor et al. [73] namely (i) **acceptability** (satisfaction, complexity, comfort, delivery and credibility of the Polypill); (ii) **adoption** (uptake, utilization, intention to try, refusal rates); and (iii) **Implementation cost** (marginal costs of the intervention and delivery strategy). Furthermore, among providers across the 12 study sites, we will assess provider satisfaction level using Healthcare provider satisfaction questionnaire [74].

## Discussion

Current projections are that the already overwhelming burden of strokes and other CVDs in Africa and other LMICs will continue to escalate over the coming decades as traditional vascular risk factors burgeon in these populations. Indeed, hypertension and dyslipidemia are the top 2 modifiable risk factors for stroke in Africa with population attributable fractions of 90.2 % and 35.8 % respectively, positioning them as prime targets for secondary risk reduction after stroke [75,76]. Identifying strategies to reduce vascular risk in LMICs will meet a global goal of reducing chronic disease death rates by an additional 2 % per year, with only a moderate rise in health expenditure [77]. Stroke is a leading cause of death, disability and dementia in most African countries, confronted with profound paucity of human resources for healthcare coordination for stroke survivors, especially in the implementation of evidence-based secondary prevention therapies.

Therefore, the proposed study would deploy a hybrid study design to 1) demonstrate the efficacy of a polypill (Polycap ^®^) containing fixed doses of antihypertensives, a statin, & antiplatelet therapy taken as two capsules, once daily orally in reducing composite vascular risk over 24 months vs. usual care among 500 recent stroke patients encountered at 12 hospitals in Ghana; 2) develop an implementation strategy for routine integration and policy adoption of Polypill for post-stroke cardiovascular risk reduction in an under-resourced system burdened by suboptimal care and outcomes. SMAART II would be premised on the feasibility and relative safety of the Polycap ^®^ for ischemic stroke among Ghanaians in a single center trial (SMAART I). This study would seek to definitively assess the efficacy and safety of the polypill to improve meaningful post-stroke global risk factor control in a larger sample, across several sites, across diverse healthcare settings, beyond tertiary level care, and over a longer period. In addition to assessing clinical outcomes, SMAART II will assess implementation outcomes pertinent to uptake of the Polypill strategy in SSA to inform policy. Regardless of its outcome, findings from SMAART II will contribute meaningful information from the African perspective to inform the formulation of guidelines for global adoption of polypills into routine care for secondary CVD risk prevention by international bodies such as the World Health Organization.

## Supplementary Material

Supplementary Table 1

Supplementary materials

Supplementary material associated with this article can be found, in the online version, at https://doi.org/10.1016/j.neuros.2026.100020.

## Figures and Tables

**Table 1:** SMAART II theoretical frameworks/models.

Model/Theory	Description	Justification

Transtheoretical	Behavioral interventions are most effective for people at the “determination” or “action” stage [52,53].	An intervention in the context of a recent stroke is likely to motivate individuals to be ready to change
Consolidated Framework for Implementation Research (CFIR) [54–57]	CFIR, a comprehensive framework for assessment of baseline, process and final implementation includes five major domains (*intervention characteristics, outer setting, inner setting characteristics of individuals and process*) with 39 underlying constructs and sub-constructs that can potentially influence efforts to change the practice. The constructs can be used as implementation and evaluation criteria in three different ways: they may (1) raise awareness for potential influential factors, (2) facilitate the analysis of pivotal processes and outcomes and (3) help organize all findings of an implementation process to explain the outcomes (i.e., to understand what worked where and why)	CFIR will be used in the pre-implementation and implementation phases of SMAART-II to capture implementation processes. This will include planning, engaging and evaluating the intervention characteristics of the polypill strategy, examining inner and outer setting barriers and facilitators, individual barriers and facilitators, as well as service and implementation outcomes of institutions, organizations, communities, and policy. The CFIR framework will guide the evaluation of implementation outcomes in the SMAART-II trial
Normalization Process theory (NPT) [58–60]	NPT is a theoretical framework that explains how new interventions, ways of acting, and ways of working become routinely embedded in everyday practice and has applications in the study of implementation processes. NPT provides a set of sociological tools to understand and explain the social processes through which new or modified practices of thinking, enacting, and organizing work are operationalized in healthcare and other institutional settings.	NPT application will help specifically with testing, implementation, embedding, & implementation of Polypill for secondary prevention after stroke into healthcare in Ghana. Focus on the NPT will be on factors that support or influence “normalization” of the integration of this intervention into routine practice in institutional settings and systems.

**Table 2:** SMAART II Trial Protocol- schedule of enrollment, intervention, and assessments.