# Supplementary material for: Stroke minimization through additive anti-atherosclerotic agents in routine treatment (SMAART) II: Rationale for a multi-country polypill phase 3 trial in sub-Saharan Africa
Source: Equity Neurosci. Author manuscript; Available in PMC 2026 Feb 24. (PMC12922694; doi:10.1016/j.neuros.2026.100020)
Supplement: Supplementary Table 1 [file NIHMS2141291-supplement-Supplementary_Table_1.docx]

**Supplementary Table S1. *Effect of Polypill on CVD outcomes for primary and secondary prevention***

| **Name of Study** | **Countries** | **LMICs site included in trial** | **Trial design** | **Study population** | **Active Intervention** | **Comparator(s)** | **Outcome measures** |
| --- | --- | --- | --- | --- | --- | --- | --- |
| **TIPS-3 ^44^**  ***Primary prevention*** | Bangladesh, Canada, Colombia, India, Malaysia, Philippines, Tanzania, Tunisia | Yes | 2 x 2 x 2 factorial RCT | Primary prevention among participants without CVDs with an elevated INTERHEART Risk Score | Polypill: 40mg simvastatin, 100mg atenolol, 25mg hydrochlorothiazide, 10mg ramipril and 75mg Aspirin | Aspirin (75mg) or  Placebo daily or  Vitamin D | Primary outcome: Deaths from CVDs occurred in 4.4% who received Polypill + Aspirin vs 5.5% in placebo, HR of 0.79 (0.63-1.00) |
| **HOPE-3 ^45^  *Primary prevention*** | 21 countries on six continents | Yes | 2 x 2 factorial RCT | Primary prevention among participants without CVD with intermediate risk of major CVDs | Polypill: 10mg rosuvastatin, 16mg candesartan and 12.5mg hydrochlorothiazide | Placebo  Lipid-lowering alone  BP lowering alone | Primary outcome: CV death/MI/Stroke occurred in 3.6% vs 5.0% in placebo, HR of 0.71 (95% CI: 0.56-0.90) |
| **PolyIRAN^48^**  ***Primary &***  ***Secondary***  ***prevention*** | **Iran** | Yes | 2-group, cluster RCT | Primary and secondary prevention among participants with and/or without CVDs | Polypill: 20mg Atorvastatin,  12.5mg hydrochlorothiazide, enalapril 5mg/valsartan 40mg, aspirin 81mg | Lifestyle advice | Primary outcome: Major CV events death /MI /Stroke occurred in 5.9% vs 8.8% in placebo, HR of 0.66 (95% CI: 0.49-0.75). |
| PROPS ^46^  ***Secondary prevention*** | U.K. | No | 1:1 parallel RCT | Secondary prevention of CVD after stroke or TIA (>55 years) | Polypill: 20mg Atorvastatin, 5mg ramipril, Aspirin 100mg | Usual care | **Yet to report study outcomes.**  BP, Lipids, Adverse effects and CV events, QOL |
| SECURE ^47^  ***Secondary prevention*** | Spain, Italy, Germany, France, Hungary, Poland, Czech | No | 1:1 parallel Open-label RCT | Secondary prevention of CVD in **elderly (>65 years)** | Polypill: 20mg/40mg Atorvastatin, ramipril 2.5, 5 or 10mg; and Aspirin 100mg | Individual lipid lowering, antiplatelet and RAAS blockers | **Primary outcome:** cardiovascular death, nonfatal type 1 MI, nonfatal ischemic stroke, or urgent revascularization occurred in 9.5% in the polypill arm vs 12.7% in the active comparator arm, HR of 0.76 (95%CI: 0.60 – 0.96). |

CVD= cardiovascular disease; HR: Hazard ratio; LMICS = Low- and Middle-Income Countries; RCT = Randomized controlled trial; TIA= Transient ischemic attack; MI= Myocardial infarction; RAAS= Renin angiotensin aldosterone system,
